# Diffusion-Controlled Drug Release from Electrospun Poly(3-hydroxybutyrate) Fibers with Beaded Architecture: An Experimental and Modeling Study

**DOI:** 10.3390/ijms27125189

**Published:** 2026-06-08

**Authors:** Alexey Iordanskii, Pavel Borovikov, Valentina Siracusa, Anatoliy Olkhov, Polina Tyubaeva, Sergey Frolov, Alexander Berlin

**Affiliations:** 1 Semenov Federal Research Center for Chemical Physics Academy of Science, Kosygina St. 4, 119991 Moscow, Russia; aljordan08@gmail.com (A.I.); aolkhov72@yandex.ru (A.O.); smfrol@chph.ras.ru (S.F.); berlin@chph.ras.ru (A.B.); 2 National Medical Research Center for Obstetrics, Gynecology and Perinatology Named After Academician V.I. Kulakov of Ministry of Healthcare of Russian Federation, 117997 Moscow, Russia; borpi@mail.ru; 3 Department Chemical Science (DSC), University of Catania, Viale A. Doria 6, 95125 Catania, Italy; 4 Academic Department of Technology and Chemistry of Innovative Materials, Plekhanov University of Economics, Stremyanny Per. 36, 117997 Moscow, Russia

**Keywords:** electrospinning, electrospun fibers, poly(3-hydroxybutyrate) (PHB), drug delivery, diffusion-controlled release, beads-on-string morphology, mathematical modeling, fiber morphology, biodegradable polymers

## Abstract

The global transition from petrochemical to sustainable bio-based plastics has been strongly supported by electrospinning (ES), a versatile nanotechnology enabling the fabrication of ultrathin fibers with multifunctional properties. The solution ES process alongside the uniform fibers, a characteristic “beads-on-string” morphology, consisting of alternating cylindrical and spindle-like segments, is frequently observed. Once considered undesirable, these structures are now recognized as functional fibrous architectures with enhanced properties. This work explores the valorization of beaded fibers through combined experimental characterization and modeling, aiming to evaluate the impact of beading on drug diffusion and delivery performance. Poly(3-hydroxybutyrate) (PHB) was selected as the model biopolyester and dipyridamole (DPD) as the model drug. Ultrathin fibers were fabricated using the laboratory electrospinning device, EFV-1 (ICP, Moscow, Russia). The distance between the capillary nozzle and the anodic collector was set to 180 mm, with the capillary tip radius equal to 0.35 mm, and applied voltage between the electrodes was kept constant at 18 kV. Drug release profiles were obtained by simulating DPD diffusion in ellipsoidal (beads) and cylindrical fiber domains. Ultrathin fibers were fabricated by solution electrospinning under environmental conditions (at ambient temperature, 50% relative humidity). Morphology was analyzed via SEM, thermal properties via DSC, and structure via FTIR spectroscopy at different temperatures, including the melting point (~170 °C). Drug release kinetics were monitored using a UV-Vis spectroscopy. The impact of DPD diffusion within the ellipsoidal and cylindrical constituents of polymer filaments was considered to modulate release profiles for the development of innovative pharmaceutical platforms. Diffusion controlled drug release was computationally modeled using a specially designed simulation program, in good agreement with experimental data. The results demonstrate that morphological parameters significantly affect diffusion and release kinetics. The controlled exploitation of bead-on-string architectures may enable the design of electrospun materials with tunable absorption of pollutant filtration, mechanical performance, and flexibility in drug release profiles, for sustainable biopolymers like PHB.

## 1. Introduction

In the 21st century, electrospinning (ES) emerged as an extremely promising and versatile nanotechnology for the fabrication of ultrathin polymer fibers. Its rapid development has been driven by continuous advances in both experimental techniques and theoretical understanding, enabling the production of functional nanofibrous materials with tailored properties [1,2,3,4]. Originally developed in the mid-20th century for filters development, electrospinning has progressively evolved into a key platform for the fabrication of biomimetic and multifunctional materials, with broad relevance in the biomedical, environmental, and industrial fields [5,6,7,8,9].

In standard electrospinning (ES), the coupling between electrostatic and viscoelastic forces represents the main physical mechanism governing the fabrication of ultrathin fibers. The electrostatic field, generated by high-voltage electrodes, together with the rheological properties of the polymer solution, determines the geometry and dynamics of the liquid jet extending between the device’s electrodes and, consequently, the quality of the resulting solidified fibers. Viscoelastic forces arise from the energy dissipation associated with molecular friction within the moving polymer jet. These forces help maintain jet integrity and limit distortions in fiber geometry by counteracting excessive jet thinning, waviness, and breakage [10]. However, an imbalance between viscous, electrostatic, and surface tension forces can lead to perturbations in the cylindrical fiber morphology, for example due to waviness instabilities during jet elongation [11,12]. Despite the relative simplicity of the experimental setup, the theoretical description of electrospinning involves a complex interplay of multiple coupled physical processes that significantly influence the geometry, morphology, crystallinity, and nanoscale structure of the fibers. Current theoretical models and computational studies have focused on the effects of key phenomena, including the non-Newtonian rheology of polymer melts and solutions [13], the electrostatic control of fiber surfaces [14], the coupling between hydrodynamic and electrodynamic forces mediated by surface tension [15,16], Taylor cone formation [17,18], as well as the heat and mass transfer processes occurring during fiber solidification [19,20].

Fiber geometry, and in particular fiber diameter as a key characteristic of electrospun materials, is strongly influenced by multiple operating parameters of the electrospinning process [21]. Under the applied electric field, the droplet located at the spinneret tip transforms into a Taylor cone, where the balance between electrostatic repulsion and surface tension governs the deformation of the initial polymer droplet and, consequently, determines fiber uniformity and diameter. An imbalance between these forces, for instance when electrostatic forces exceed surface tension, can result in imperfections such as whipping, bending, and bead formation [22,23,24].

In addition to these primary parameters, several secondary factors contribute to the overall quality of fibrous materials, including single filaments, mats, and functional porous membranes. The integration of electrospinning modeling with extensive experimental data allows the identification of optimal processing windows for both petrochemical and bio-based ultrathin polymers, thereby supporting the transition from laboratory-scale production to large-scale manufacturing.

Nevertheless, due to jet instability and the influence of both internal and external parameters, the fabrication of electrospun fibers with uniform architecture remains challenging and often requires empirical optimization. For example, deviations in polymer viscosity from optimal values or insufficient applied voltage can lead to beads-on-string morphologies or even the formation of isolated droplets [25].

Beaded fibers are frequently observed and have traditionally been regarded as defects that negatively affect mechanical performance, filtration efficiency, diffusion behavior, and biomedical functionality. A comprehensive analysis of electrospinning parameters responsible for bead formation has been recently reported in the literature [26], where beading is still primarily considered an undesirable phenomenon to be minimized or eliminated.

Very recently, electrospun fibers with a beads-on-string architecture have attracted increasing attention as promising fibrous systems with advanced functionality. Indeed, the perception of beaded structures is evolving, recognizing them as unique constructs with functional properties that differ from those of conventional cylindrical fibers. For instance, beaded morphologies, commonly observed in electrospinning, have been successfully used for the design of superhydrophobic polymer surfaces by Rabello et al. [27]. Rasouli et al. [28] investigated polysulfone (PS) beaded fibers obtained by varying processing parameters and solution characteristics, showing that under critical electrospinning conditions a transition from spherical to ellipsoidal geometries can occur. The electrohydrodynamic printing mechanism has also been shown to involve the formation of secondary beads embedded within the polymer jet. Furthermore, the nature and behavior of beaded ultrathin fibers have been comprehensively analyzed by Wang et al. [29]. One of the pioneering studies on the application of beaded fibers as carriers for therapeutic agents was recently reported by Chen et al. [30], who demonstrated that bead formation enables modulation of drug release kinetics, reducing the burst effect and extending sustained release due to increased drug loading capacity. While providing a brief overview of the positive effects of beaded fibers in applications such as filtration [31] and controlled delivery [32], it is also important to mention Janus particles, characterized by their dual nature, which enable the design of two-stage therapeutic systems with versatile kinetic profiles [33]. More recently, a significant study explored the immobilization of polymersomes as antibody carriers for transmucosal therapy [34].

The topology and morphology of fibrous carriers play a crucial role in regulating the magnitude and direction of diffusion fluxes, thereby defining the spatiotemporal distribution of bioactive compounds. This, in turn, affects pharmacokinetics, food shelf life, and environmental remediation processes. In most studies on delivery performance, diffusion is typically modeled as transport in uniform cylindrical fibers with variable morphology, crystallinity, diameter, and porosity. Such fibrous systems are characterized by a high surface-to-volume ratio like for foams, nanoparticles, and micelles. However, electrospun fibers differ significantly in geometry and morphology from other delivery platforms such as bulk devices and macro prostheses, offering advantages in terms of therapeutic efficiency, local targeting, and reduced toxicity, in biomedical and environmental applications [32]. The combination of features typical of nanofibers and particulate systems provides a basis for the development of innovative therapeutic platforms with tailored release profiles. In this context, beads-on-string architectures in electrospun fibers loaded with bioactive compounds (BCs) can enhance such synergistic effects, provided that diffusion processes in these geometrically complex systems are properly understood.

To deepen the understanding of transport phenomena in such architectures and to optimize diffusion-controlled delivery, it is essential to adopt a comprehensive approach combining experimental investigation and theoretical modeling. This dual strategy enables a more accurate description of transport dynamics in fibers with complex geometry and supports the development of more efficient functional materials. The selection of poly(3-hydroxybutyrate) (PHB) as a model system is motivated by its sustainability, excellent ES processing, favorable mechanical properties, and controlled biodegradability, as well as by our previous experience in studying transport phenomena in this biopolyester. In particular, diffusion and structural evolution have been previously investigated in PHB-based systems, including drug-loaded films [35], nanoparticles [36], and uniform ultrathin fibers [37], considering also segmental mobility [38], hydrolysis [39], and biodegradation processes [40].

In view of emerging challenges in biomedicine, packaging, and environmental safety, electrospun fibers with beads-on-string architecture are investigated in this work at both the experimental and modeling levels. In the present study, PHB fibrous carriers loaded with a BC were examined to evaluate the effect of thickened beads, simulated as ellipsoids, on release performance, representing the main novelty of the paper. In addition, the influence of the geometrical features of beads-on-string structures was analyzed to predict the impact of polymer morphology on diffusive transport under different topographical conditions.

To the best of our knowledge, this is the first study addressing diffusion-controlled drug release from electrospun fibers with beads-on-string architecture through a combined experimental and computational modeling approach.

## 2. Results and Discussion

### 2.1. SEM of PHB Electrospun Fibers for Beads-on-String Modeling

**Figure 1:**
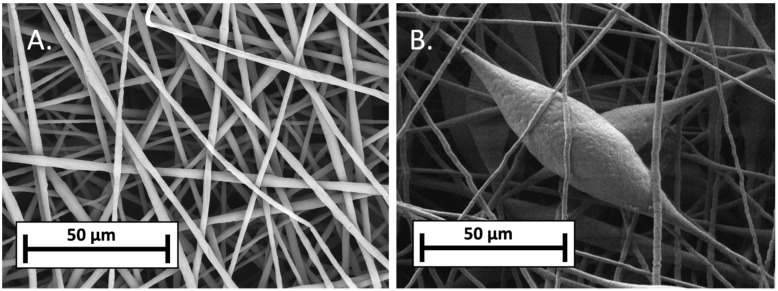
SEM images of PHB (bar 50 μm, magnification ×4000): electrospun fibers characterized by cylindrical geometry (**A**); electrospun fibers characterized by presence of thickenings approximated by a prolate ellipsoid (**B**).

As result of electrospinning, ultrathin fibers often contain specific thickenings in the form of beads with variable geometry, ranging from spherical particles to spindle-like structures. Similar morphologies have been reported in numerous studies for both synthetic and natural polymers [29,41]. In this context, biodegradable plastics, due to their sensitivity to electrospinning conditions, frequently exhibit a beads-on-string architecture [42,43]. Among the key causes of structural element formation in the form of beads, the following are generally considered: the viscosity of the spinning solution, determined by the molecular weight of the polymer [44], the applied voltage, and the jet length of the polymer solution [45], and processing conditions, particularly humidity [46,47]. When the balance of process parameters is maintained, it is possible to obtain smooth uniform fibers (Figure 1A). A shift in one or several parameters leads to the formation of characteristic cylindrical beads (Figure 1B). It should be noted that varying the solution viscosity by altering either the molecular weight of the polymer or the mass concentration of the polymer in the solution allows for changing the bead shape from spherical to ellipsoidal [48]. Voltage control allows for obtaining thinner fibers in the presence of elliptical beads, which can be effectively used for drug delivery, as they are predominantly represented by amorphous regions of PHB where additives are typically localized, as demonstrated by Tyubaeva et al. using the PHB–chlorophyll system as an example [48]. As shown in Figure 1, for 7 wt% PHB in CL with molecular weight 4.6 × 10^5^ g/mol under various ES parameters (voltage 18 kV, tip-to-collector distance 180 mm and voltage 22 kV, tip-to-collector distance 220 mm for A and B, respectively), it is possible to obtain characteristic “containers for drug loading”.

In PHB, the alternating fragments of the beaded fibers, namely uniform cylindrical fragments (Figure 1A) and ellipsoid-like thickenings (Figure 1B), form the overall morphology of the electrospun biopolymer mat with randomly distributed beads. These structures serve as the basic model for evaluating the kinetic profiles of BC (DPD) release, and their shape can be approximated by geometrical forms such as a prolate ellipsoid of rotation.

### 2.2. Beads-on-String Architecture Modeling

**Figure 2:**
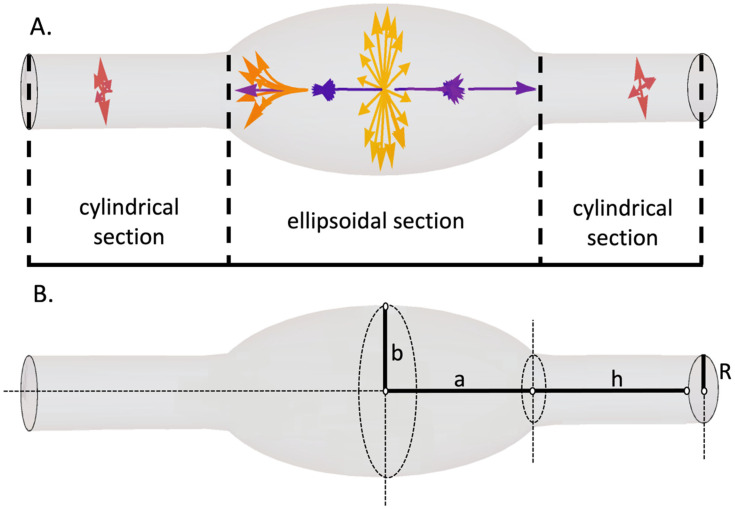
Schematic representation of BC flux vectors directed toward the ellipsoid boundary during release (**A**). Schematic representation of the fiber architecture used for modeling BC delivery, where a and b are the major and minor semi-axes of ellipsoid, respectively; h is half of distance between adjacent ellipsoids, and R is the radius of the electrospun fiber (**B**).

Schematic representation of the beads-on-string fibrillar architecture is shown in Figure 2. It should be noted that the release process involves the mass transfer of the BC, which can be represented as vectors directed from the center of the fiber to its boundary [49]. The visualization of BC mass transfer within a cylindrical fiber section and an ellipsoidal fiber section is presented in Figure 2A. For the mathematical modeling of mass transfer kinetics, it is proposed to introduce the semi-axes (a, b) and the average distance (2 h) between two adjacent ellipsoids located along the same filament (as on a string), as fixed values for modeling the rate of BC release is determined by its diffusivity in the PHB matrix (Figure 2B).

Parametric representation of points within volume and surface of cylindrical and ellipsoidal sections using prolate spheroidal coordinates were [50]:

x = F_0_ sinh (r) sin (θ) cos (φ), y = F_0_ sinh (r) sin (θ) sin (φ), z = F_0_ cosh (r) cos (θ), (1)

where 0 ≤ r < +∞, 0 ≤ θ < π, 0 ≤ φ < 2π, and 0 ≤ F_0_ < +∞; sin (θ) and cos (φ) denote the trigonometric sine and cosine functions, while sinh (r) and cosh (r) represent the hyperbolic sine and cosine functions, respectively.

2F_0_ is the distance between the foci of the ellipsoid, while a and b are its major and minor semi-axes, respectively. These parameters are related through the standard relation for the foci:

(2) $${F_{0}}^{2}=a^{2}-b^{2}.$$

### 2.3. Diffusion-Controlled BC Delivery: Modeling of Beads-on-String Fiber Geometry

Diffusion-controlled release of BA in the three-dimensional space of an ellipsoid is described by:

(3) $$\frac{\partial C}{\partial t}=D\nabla^{2}C$$

where C is the concentration of BA at any point in the PHB matrix at time t, ∇ is the differential operator in ellipsoidal coordinates, and D is the isotropic diffusion coefficient.

For an ellipsoid homogeneously loaded with BA (DPD), the initial and boundary conditions at the onset diffusion-controlled release are defined as:

(4) $$C\left(x,y,z,0\right)=C_{0}\;\;at\;t=0$$

and at the ellipsoid boundary for all times of diffusion:

(5) $$C\left(x_{0},y_{0},z_{0},t\right)=0\;\;\mathrm{at}\;t>0$$

The combination of Equation (3) with the auxiliary conditions (4) and (5) lead to the general form:

(6) $$C\left(x,y,z,t\right)=\Phi \left(x,y,z\right)\exp\left(-\frac{c^{2}Dt}{{F_{0}}^{2}}\right)$$

where the function Φ satisfies the Helmholtz equation:

(7) $$\left(\nabla^{2}+\frac{c^{2}}{{F_{0}}^{2}}\right)\Phi =0$$

where c is the eigenvalue obtained through separation of variables.

To solve Equation (7) for the ellipsoidal geometry, the spatial coordinates (r,θ,φ) are introduced according to Equation (1), together with the following notation:

(8) ξ=cosh(r),η=cos(θ),ζ=cos(φ)

If the initial and boundary conditions do not depend on φ, the solution is also not dependent on φ. Under axial symmetry, this condition is expressed as:

∂/∂φ = ∂/∂ζ = 0,(9)

which simplifies the diffusion problem. Additional intermediate steps of the derivation are provided in the Supplementary Materials.

The solution of the diffusion equation governing BA release is then expressed as:

(10) $$C\left(\xi ,\eta ,t\right)=\sum_{n=0,2,\ldots }\sum_{k=1}A_{nk}\exp\left(-\frac{{c_{nk}}^{2}Dt}{{F_{0}}^{2}}\right)R_{n}\left(c_{nk},\xi \right)S_{n}\left(c_{nk},\eta \right)$$

And at t=0:

(11) $$C_{0}\left(\xi ,\eta ,0\right)=\sum_{n=0,2,\ldots }\sum_{k=1}A_{nk}\;R_{n}\left(c_{nk},\xi \right)S_{n}\left(c_{nk},\eta \right)$$

The algorithm used to evaluate the coefficient $A_{nk}$ is provided in the Supplementary Materials.

Knowing the concentration distribution from Equation (10), it is possible to find the total amount of BA released, by integration of over the ellipsoid volume V:

(12) $$M\left(t\right)=\int_{V}C\left(r,\theta ,\varphi ,t\right)\mathrm{d}V,$$

where M(t) is the cumulative amount of BC remaining in the ellipsoid at time *t*.

The proposed mathematical model allows for the consideration of key mass transfer aspects within two main structural elements of the fiber: cylindrical and ellipsoidal. The calculation obtained could be applied to the modeling of controlled drug delivery, which is discussed below.

Computerized approaches commonly synthesize a large amount of information acquired about the evolution of the polymer system state (time as a parametric characteristic) and obtained from a complex of comprehensive experimental methods. Therewith, the inherently modeling process is never a completely terminated representation of a biopolymer’s evolution under exploitation. Under the impact of new and reliable results, an essential discrepancy may have arisen between the model and experimental findings. In a way, this discrepancy can be beneficial for the enhancement in our academic and industrial concepts. However, until thorough studies yield a convincing set of new facts that contradict the conclusions drawn from the model, we should continue to use the existing modeling framework as a fruitful compass with the predictive availability.

### 2.4. Impact of BC Diffusivity on Delivery Kinetics

**Figure 3:**
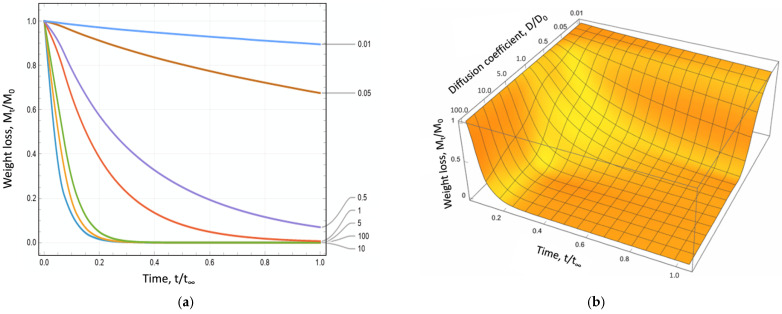
Effect of BC diffusivity on the release kinetic profiles, presented in dimensionless coordinates: time (t/t_∞_) and BC weight loss (M_t_/M_0_) (**a**). Effect of BC diffusivity on release profiles in three-dimensional dimensionless coordinates: time (t/t_∞_), BC mass loss (M_t_/M_0_), and diffusion coefficients (D/D_0_) (**b**), where M_0_ is the initial amount at t = 0, and D_0_ is the reference diffusion coefficient, equals to 1.0 × 10^−10^ cm^2^/s.

Using the diffusion equation for BC delivery presented above (Equation (3)), together with the corresponding boundary (Equation (4)) and initial (Equation (5)) conditions, the proposed model clearly demonstrates the significant influence of the diffusion coefficient D (expressed as the dimensionless parameter D/D_0_) on the kinetic profile of drug release. The critical role of diffusion in drug release from ultrathin PHB fibers has been previously demonstrated using a simplified model [37]. In that study, diffusion within the ellipsoidal domains was not considered due to the predominantly cylindrical morphology of the electrospun fibers. Moreover, the low concentration of ellipsoid-like thickenings formed during fiber solidification was neglected in the analysis. As a result, diffusion was assumed to govern drug release exclusively from cylindrical fibers. Furthermore, for electrospun fibrous biopolymers such as polylactic acid (PLA), polycaprolactone (PCL) and polyvinylpyrrolidone (PVP), the significant impact of diffusion on drug release kinetic has been widely reported [51,52,53,54,55,56]. However, in the existing literature, drug release from ellipsoid-like architectures has not been explicitly addressed and is typically regarded as a consequence of structural defects in electrospun fibers. In the present study, the role of diffusion in the overall delivery process from bead-on-string structures is first illustrated in Figure 3, which depicts the mobility of BC in two- and three-dimensional coordinates, (a) and (b) respectively.

It is well known that diffusion is a topologically controlled process, i.e., it depends on the geometry of the polymer medium. Both the geometrical shape and morphology determine the length of the diffusion path along which the BC release flux propagates. For an ellipsoid, the key parameter determining the shortest diffusion path is its minor semi-axis (b). Therefore, it is relevant to further examine the role of the geometrical parameters of the model structure shown in Figure 2B. It is worth noting that the SEM imaging of the PHB ellipsoid (Figure 1B) allows the identification of the characteristic geometrical parameters of the beaded fibrous system, namely *a* = 17 µm, *b* = 7.2 µm, and cylinder radius R = 1.5 µm.

The motivation for the diffusion simulation is further supported by the fact that the beaded ultrathin fibers with ellipsoidal geometry are capable of encapsulating proteins and cells and can favorably influence the burst effect occurring during the initial stage of BA release [57]. This beneficial behavior of the beads-on-string architecture promotes the use of electrospun fibers in tissue engineering as functional scaffolds [58,59] and in the design of therapeutic platforms with prolonged release profile [30,60]. Furthermore, for the encapsulation of high-molecular-weight BC, such as peptides and proteins [59,60,61], carotene [62], DNA [63], and polymersomes [34], the available volume of the polymeric carrier—specifically the size and axes of the ellipsoidal thickenings—must be sufficient to accommodate these large molecules.

**Figure 4:**
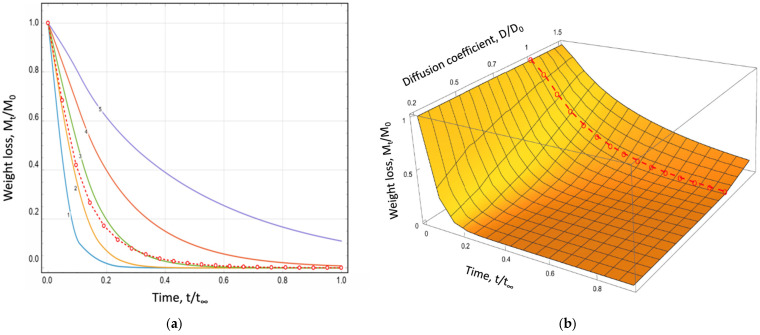
Simulated release profiles for the beads of different geometries: cylinder (1), ellipsoids (2–4) and sphere (5) (**a**), where the numbers of the curves indicate the corresponding values of the ratio R/b: (1) 0.2, (2) 0.5, (3) 0.7, (4) 1.0, and (5) 1.5. Three-dimensional representation illustrating the effect of the ellipsoid minor semi-axes on BC release profiles (**b**), where the red dotted curves corresponds to experimental data for the DPD-PHB system.

To investigate the impact of ellipsoidal geometry at the micrometer scale on the delivery kinetic of BA (DPD), computational modeling was performed for different values of the minor semi-axes (b), expressed through the dimensionless ratio R/b. In Figure 4, the lowest kinetic curve (1), corresponding to release from cylindrical fibers only, and the upper curve (5), corresponding to BA release from single spheres as another limiting case of beads-on-string architecture, represent the extreme release profiles for cylindrical and spherical geometries, respectively. The intermediate curves (2–4) describe the cumulative release from combined ellipsoidal and the cylindrical domains, with varying *R/b* values reflecting the relative contribution of diffusion paths in the spherical and cylindrical regions.

The variation in the ratio R/b clearly shows that, at the same diffusivity (1.04 × 10^−10^ cm^2^/s), the rate of BC delivery increases in the following sequence: sphere < ellipsoid < cylinder. This trend is primarily determined by the topology of the polymeric drug carriers and suggests that the presence of specific thickenings (beads or spindle-like structures) in the form of ellipsoids with different minor semi-axes (b) may promote a more pronounced sustained release effect, potentially leading to improved therapeutic performance at both molecular and cellular levels.

**Figure 5:**
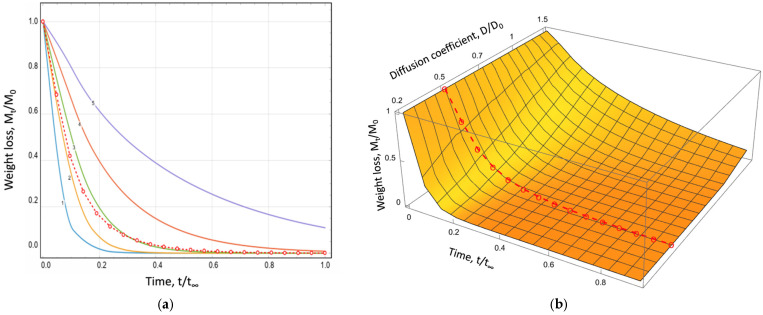
Effect of the distance between the neighboring ellipsoids (2 h) located along the same string. (**a**): Simulated delivery curves showing their evolution at different a/h ratios: (1) 0.071, (2) 0.11, (3) 0.18, (4) 2.5, (5) 3.1. (**b**): Three-dimensional representation of BC release kinetic profiles. Here, *a* and *b* denote the major and minor semi-axes of the ellipsoid, respectively, and D is the diffusion coefficient of the BC–polymer system, where the red dotted curves corresponds to experimental data for the DPD-PHB system.

Figure 5 illustrates a realistic situation in which the density of ellipsoid-like structures increases, corresponding to a decrease in the a/h ratio. It follows from the figure that, at a critical ratio between the major semi-axis of the ellipsoid (a) and the distance between adjacent ellipsoids (h), namely when h > a, the contribution of diffusion from the cylindrical regions of the fiber begins to dominate over that from the ellipsoidal domains. Under this condition, the BC release rate increases significantly (curves 1–3), as diffusion through the cylindrical surface of the fiber becomes the governing process.

### 2.5. DSC and FTIR Characterization of PHB Fibers Containing BC

**Figure 6:**
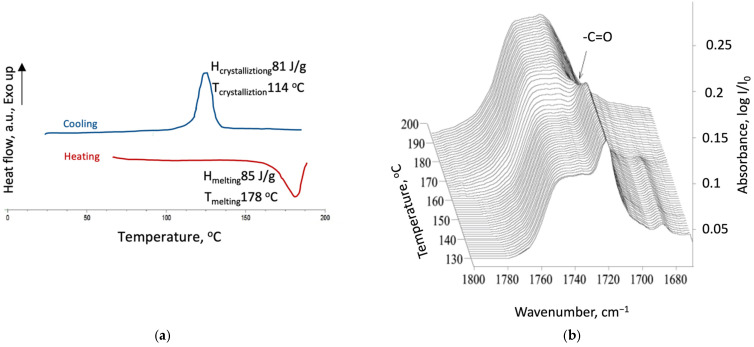
DSC curves of the PHB fibrous mats recorded during heating and cooling (**a**); FTIR spectra as a function of temperature in the region of the -C=O stretching vibration (**b**).

Figure 6a shows the typical DSC thermograms of PHB ultrathin fibers recorded during heating and cooling. The DSC curves exhibit endothermic and exothermic phase transitions associated with the melting and crystallization of the fibrous material, respectively. Table 1 reports the degree of crystallinity for PHB samples loaded by different DPD content calculated from the melting enthalpy. The results obtained suggest that the encapsulated DPD influence the crystallization process primarily during the solidification stage of the polymer jet. However, after fiber formation, at temperatures below the melting point, the interactions between DPD and PHB appear to be weak, as indicated by the consistent melting temperature values, which remain essentially unchanged regardless of the polymer–BC system composition.

Changes in PHB segmental mobility during heating and cooling have been previously observed by the authors using the ESR spin-probe technique [64], as well as by DSC measurements, where the corresponding phase transitions were identified (see Figure 6). In addition, further insight into the thermal behavior of the biopolyester were obtained by recording FTIR spectra at different temperatures. Figure 6b presents a series of FTIR spectra recorded for heated PHB fibrous samples. As shown in the figure, the spectral bands at approximately 1724 ± 2 and 1280 ± 2 cm^−1^ are attributed to C=O stretching and asymmetric C–O–C stretching vibrations, respectively, associated with the ester bonds of the PHB backbone [65]. It is important to note that the carbonyl stretching band of the ester group in PHB is highly sensitive to changes in polymer chain mobility. The increase in segmental mobility is associated with the melting of PHB crystallites, which occurs over a relatively narrow temperature range. A low-frequency shoulder of the C=O band (around 1724 cm^−1^) disappears near the melting temperature of PHB, and above ~172 °C, the polymer melt exhibits a single broad band with a maximum at approximately 1740 cm^−1^. The broadening of this band likely reflects a wide distribution of mobile conformations contributing to the vibrational behavior of ester groups in the melt. Notably, the temperature corresponding to this change in molecular mobility, as indicated by FTIR data, is in good agreement with the melting temperature obtained from DSC measurements. Thus, a clear correlation is observed between FTIR spectral changes and DSC thermograms, both indicating the melting phase transition in the same PHB samples used as DPD delivery systems.

The observed increase in the degree of crystallinity of PHB with increasing DPD content is associated with a decrease in the density of ellipsoidal structures. It has been previously demonstrated that the incorporation of the drug into the polymer solution used for electrospinning enhances its electrical conductivity, which in turn reduces bead formation in the fibers. As a result, within the framework of the proposed model, the h/a ratio increases, thereby diminishing the influence of the beads-on-string architecture on PHB crystallinity. During the electrospinning process, the residual solvent (CL) evaporates from the liquid jet into the surrounding environment. Under diffusion-controlled desorption, the rate of solvent loss is proportional to the square of the cylindrical fiber radius and to the square of the minor semi-axis of the ellipsoids, representing competing processes. Therefore, the ratio of characteristic times for solvent removal from cylindrical and ellipsoidal domains can be expressed as proportional to (R/b)^2^. Accordingly, the rate of solvent evaporation from the cylindrical regions of the fibers is expected to be significantly higher—by up to two orders of magnitude—than from the ellipsoidal domains. As a consequence, beads-on-string structures may retain residual solvent, which can hinder complete crystallization. An opposite trend is observed when electrospun fibers are annealed at 140 °C for 2 h under a nitrogen atmosphere. As shown in Table 1, after annealing, the crystallinity of PHB becomes independent of DPD content and approaches a constant value corresponding to its thermodynamic equilibrium in the fibers. In future work, this effect could be quantitatively described using a diffusion-based model analogous to that presented above, but accounting for higher solvent diffusivities. Due to the high volatility of CL, it is expected to rapidly migrate from the bulk of the electrospun PHB jet into the surrounding vapor phase.

### 2.6. Comparison Between Simulated and Experimental Release Kinetics

SEM micrographs, providing information on the morphology of PHB ultrathin fibers for accurate estimation of release kinetics and subsequent evaluation of drug (DPD) diffusivity, play a crucial role in supporting the application of fibrillar therapeutic systems. In this context, the fitting algorithm used to match experimental release kinetics with computational simulations should be carefully controlled to minimize deviations between experimental and modeled results. Deterministic modeling is commonly employed to reproduce release profiles based on polymer carrier properties, particularly diffusion coefficients in polymer matrices [66]. However, as recently highlighted in a comprehensive study by Yang et al. [66], such an approach typically provides “only a single point estimate, neglecting the inherent statistical variability” and stochastic deviations present in experimental data.

For the release pattern formed by the family of kinetic curves, following the approach proposed in the reference study [67], the comparison between experimental and calculated data was performed using an objective function. A simplified form of the comparison function (Fc), which was applied to determine the diffusion coefficient as the final parameter for the release profile analysis:

(13) $$F_{D}\;=\;{\sum_{i}^{N}wi(Di\left(experiment\right)-D\left(model\right))}^{2}$$

where Di and D are the experimentally observed and modeled diffusivities, respectively. The weighting coefficient, w_i_, corresponds to the reciprocal value of standard deviation and account for instrumental uncertainties and heterogeneities in the polymer structure.

**Figure 7:**
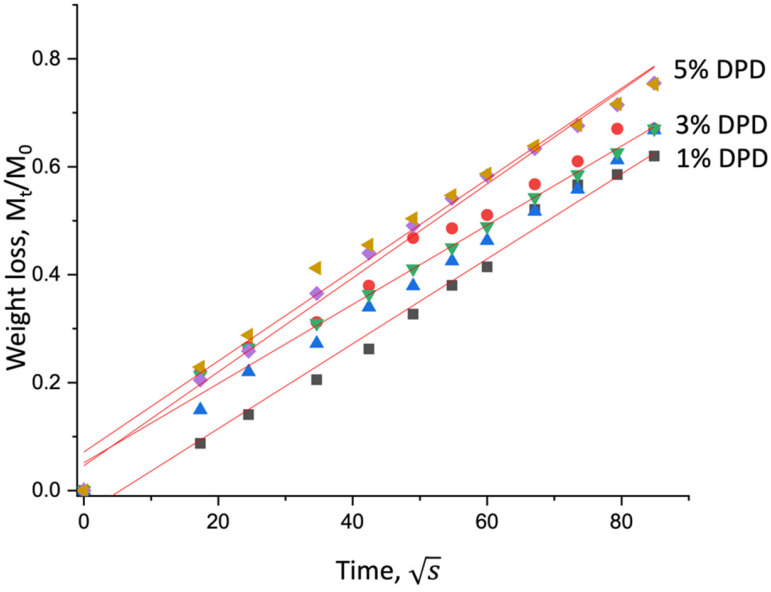
Initial stage of DPD release from PHB electrospun fibers, presented in diffusional coordinates: “weight ratio of DPD delivered vs. square root of time”. Here, M_t_ and M_∞_ are the weight of DPD released at time *t* and at *t* → ∞, respectively.

The above-mentioned study [66] further developed this approach by introducing an improved iterative procedure for data comparison. For the PHB-DPD fibrillar system, the experimentally determined release kinetic profiles at different drug loadings are presented in Figure 7. The symbols (circles, triangles) represent the experimental data obtained from UV measurements, while the solid lines correspond to the fitted curves obtained via simulations using the Origin software (version 2026 SR1).

Table 2 reports specific experimental data and statistical analyses demonstrating the reliability of the modeling approach, as well as the slight differences between instrumental data (UV spectrometry) and simulation results.

The time-evolution of the local concentration within the ellipsoidal and cylindrical domains was calculated and interpolated using the model described above, based on the general diffusion equation (Equation (2)) and the corresponding initial and boundary conditions (Equations (4) and (5)). The system of modeling equations was solved by minimizing the sum of the squares of the residual deviations for the apparent diffusivities of the drug in the polymer phase.

Numerical modeling in polymer science plays a crucial role in gaining insight into the complex, multifaceted behavior of biodegradable polymer systems. Therewith, the inherent modeling process is never a completely terminated representation of a biopolymer’s evolution. This is simultaneously both a strength and a weakness of the mathematical approach. Under the impact of new and reliable results, an essential discrepancy may have arisen between the model and experimental findings. In a way, this discrepancy can be beneficial for the enhancement in our academic and industrial concepts. However, until thorough studies yield a convincing set of new facts that contradict the conclusions drawn from the model, we should continue to use the existing framework as a fruitful compass with predictive availability.

## 3. Materials and Methods

### 3.1. Materials

The electrospun fibers were fabricated from poly(3-hydroxybutyrate (PHB) (16F series) kindly provided by Biomer Comp., (Krailling, Bavaria, Germany). The PHB was produced via microbiological synthesis and, as the final product, is characterized by a molecular weight (Mw) of 4.6 × 10^5^ g/mol and density of 1.248 g/cm^3^. PHB, in the form of fine white powder, was dissolved in chloroform (CL) (CHCl_3_, chemical grade) at elevated temperature. The use of dipyridamole (DPD) [molecular formula C_24_H_40_N_8_O_4_, NIC index: CID 3108] for the study of drug delivery from PHB fibers is attributable to its widespread clinical application, predominantly as an anticoagulant. Dipyridamole (Mw = 504.6 g/mol; purity > 98.0%; melting point 164.2 ± 1.5 °C) was supplied by Beijing Inoke Technology Co., Ltd., Beijing, China. As the model mobile compound, the DPD solution was gently added dropwise to the CL solution of PHB. To prepare the solution for subsequent electrospinning, both DPD and the polyester were dissolved in CL (0–5 wt%) under stirring at a rotation rate of 250 rpm and at 37 °C.

### 3.2. Electrospinning Technique

**Figure 8:**
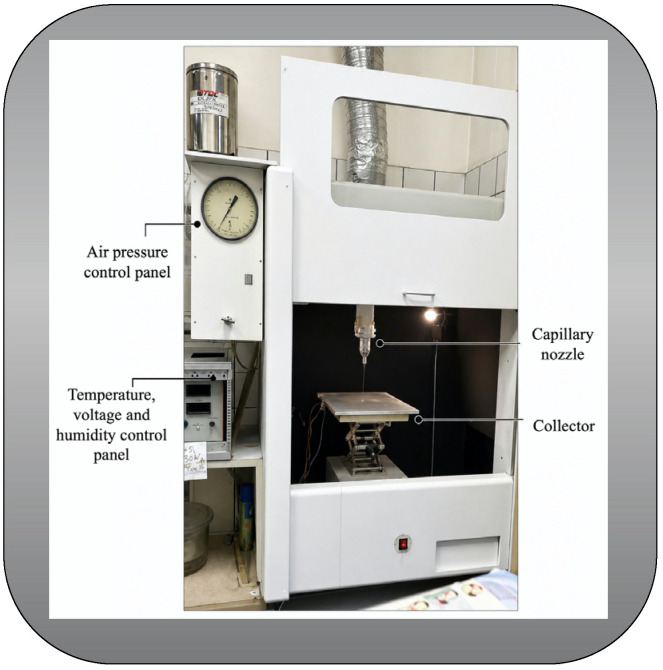
Electrospinning device used for fabrication of ultrathin polymer fibers.

Ultrathin fibers were fabricated using the laboratory electrospinning device EFV-1 (ICP, Moscow, Russia), as shown in Figure 8. The distance between the capillary nozzle and the anodic collector was set to 180 mm, which allows the formation of solidified fibers in the shape of mats. Similar distances between the capillary tip (0.7 mm diameter) and the flat collector electrode have been recently reported for other solution electrospinning setups [68]. A shorter nozzle-to-electrode distance can accelerate fiber deposition on the collector surface, resulting in limited solvent desorption time and incomplete evaporation of CL. Notably, an excessively long distance may reduce the quality of fiber deposition and packing on the collector surface [69].

The polymer solution flow rate represents another critical parameter in fiber fabrication and was maintained constant at 1.0 mL/h. An increase in the flow rate leads to the jet flow acceleration, which may cause jet instability and disintegration, accompanied by droplets formation. The applied voltage between the electrodes was kept constant at 18 kV.

The temperature and relative humidity inside electrospinning chamber were settled in the range of 23–25 °C and 50 ± 1% RH, respectively.

### 3.3. Scanning Electron Microscopy Analysis

The architecture and morphology of the electrospun beaded fibers were investigated via Scanning Electron Microscopy (SEM) using a Hitachi TM-1000 instrument (Hitachi, Tokyo, Japan) at an accelerating voltage of 20 kV. Electrospun fibers were previously sputter-coated with a gold/palladium layer of 100–200 Ǻ. The specimen size was 10 × 10 mm, and the mean diameters were calculated based on a sample of 100 fibers. Fiber diameters were statistically evaluated from SEM images using ImageJ software (version 1.54t), applying the elliptical selection tool.

### 3.4. DPD Delivery Rate Registration

The release of DPD from PHB electrospun fibers was carried out as follows: a rectangular fragment of the mat (∼10 mg) was cut and suspended in 50 mL of phosphate buffer solution (pH = 7.4 ± 0.2) at 37 °C under continuous stirring at 200 rpm in a thermostatically controlled glass flask. To determine the amount of DPD released into the phosphate buffer, 3.0 cm^3^ aliquot was withdrawn at appropriate time intervals and analyzed using a UV–Vis spectrometer (DU65 (Beckman Coulter Inc., Brea, CA, USA) at wavelengths in the range of 295–300 nm. The maximum of the UV absorption band of DPD was observed to shift within this range. To monitor the shift, the full UV spectrum was recorded every 10 min during the release experiment. Additionally, each experimental kinetic point was followed by background optical density measurement. For each aliquot removed, an equal volume of fresh buffer solution was added. Each measurement was performed in triplicate, and the averaged value was used as a single experimental point.

### 3.5. Differential Scanning Calorimetry (DSC)

Differential Scanning Calorimetry (DSC) measurements were carried out using a TA Instruments Q20 DSC model (New Castle, DE, USA). Fibrous samples as PHB mats of approximately 3 mg were cut and sealed in standard aluminum pans. The DSC cell was maintained under nitrogen atmosphere with a gas flow rate of 20 cm^3^/min. The samples were equilibrated at 40 °C, then heated up to 180 °C at a rate of 10 °C/min and held isothermally for 3 min. After this procedure, they were cooled down to 0 °C at the same rate of 10 °C/min. The melting temperature (T_m_) and the glass transition temperatures (T_g_) were determined from the peak maximum and the inflection point of the heating scan, respectively. Data were analyzed using TA Universal Analysis v4.5A software (New Castle, DE, USA). T_g_ was taken as the midpoint of the heat capacity changes. The cold crystallization temperature (T_cc_) and Tm were determined from DSC curves, while the degree of crystallinity (χc) was calculated through the following equation, as reported in [70]:

$$\frac{{\Delta H}_{m}-\Delta H_{cc}}{{\Delta H}_{m}^{c}\times \alpha_{PHB}}\times 100\%$$

where ΔH_m_ is the melting enthalpy, ΔH_cc_ is the cold crystallization enthalpy, and ${\Delta H}_{m}^{c}$ is the melting enthalpy of a hypothetically 100% crystalline PHB (105 J/g). For the homopolymer, α_PHB_ = 1. In PHB sample loaded with 1.0, 3.0, and 5.0 wt% DPD, this parameter assumes values of α_PHB_ equal to 0.99, 0.97, and 0.95, respectively.

### 3.6. FTIR Method

FTIR spectra of PHB electrospun fibers were recorded using a Bruker IFS-48 IR spectrometer (Bruker GmbH, Mannheim, Germany) in the range of 500–3500 cm^−1^ at a resolution of 2 cm^−1^; the number of scans was equal to 85. The ATR accessory was equipped with a diamond crystal to obtain effective multiple internal reflection (×7). Spectra were obtained at different temperatures at the range 130–200 °C. Spectra were analyzed using the OPUS^TM^ software (version 9.3) (Bruker GmbH).

### 3.7. Statistical Analysis

The melting enthalpy of PHB fibers was evaluated using NETZSCH Proteus (version 5.2.1). The average statistical error in thermal measurements was ±5%. The kinetic release experiments of DPD release were performed in duplicate to obtain averaged values of the diffusion curve constants, with a regression coefficient (R^2^) of approximately 0.989 for all initial kinetic release profiles. All other results (FTIR, SEM) were considered statistically significant based on Student’s *t*-test, with a significance level of *p* < 0.05. Detailed statistical data are presented in Section 2.5.

All calculations and graphical representations presented below were performed formed using Mathematica software (Version 14.2) environment.

## 4. Conclusions

Thanks to the rapid development of electrospinning as a modern nanotechnology, innovative ultrathin fibers with enhanced functionality have attracted considerable interest for applications in key areas of human activity. However, the production of ideal polymer fibers is often accompanied by structural irregularities, such as breaks, twists, and, more frequently, localized thickenings known as “bead-on-string” structures [29,41]. Until recently, such morphologies were considered undesirable byproducts associated with reduced material quality. This perspective has now changed, and “bead-on-string” architectures are increasingly recognized as unique fibrous systems with enhanced functionality resulting from the synergistic combination of microparticle-like properties and cylindrical nanofibers. This trend is particularly evident in filtration technologies addressing environmental challenges, as well as in the development of advanced therapeutic platforms.

By studying fiber morphology and diffusion phenomena, the authors aimed to exploit beaded PHB fibers as biocompatible and biodegradable carriers loaded with DPD as a model compound. PHB is currently considered one of the leading commercial sustainable biopolymers with favorable application properties. In this work, computational modeling performed using Mathematica™ software was combined with experimental data on the morphological and kinetic characteristics of PHB fibers. The interaction of DPD diffusion within the ellipsoidal and cylindrical constituents of polymer filaments was considered to modulate release kinetics for the development of innovative pharmaceutical platforms. A detailed computational analysis based on SEM micrographs was conducted to evaluate the influence of key geometric parameters, including the major and minor axes of the ellipsoids, the fiber diameter, and the distance between a pair of adjacent ellipsoids. The latter parameter reflects the density of the beads on the string in the PHB fibrous mat and plays a crucial role in governing drug release kinetics.

Despite the satisfactory consistency between experimental results and simulated results of diffusion phenomena controlling drug delivery, as well as the high suitability of the proposed model for bead-on-string architecture of electrospun fibers, several limitations must be acknowledged. First, the model only considers DPD diffusion and does not account for potential interactions with water molecules adsorbed on PHB ellipsoids from physiological or environmental media. While such interactions are particularly relevant for hydrophilic drugs, future work should focus on developing a binary diffusion model that includes cross-interactions at the molecular level. Second, the current approach is limited to bead-on-string architectures, although it could be extended to other types of morphological irregularities resulting from instabilities in the electrospinning jet.

In conclusion, the results presented, together with the literature review provided in the introduction, highlight the significant potential of ultrathin fibers with bead-on-string architectures. These complex morphologies offer valuable opportunities for the design of tailored release profiles that meet the requirements of biomedical applications [71], contribute to environmental protection [72] and support the development of environmentally friendly active packaging systems [73].

## Figures and Tables

**Table 1:** Physicochemical characterization of the PHB-DPD fibrous system.

DPD wt%	α^0^_C_ (%)	α^T^_C_ (%)	T_MP_ (°C)
0	30 ± 5	65 ± 8	171 ± 14
1	38 ± 5	68 ± 7	168 ± 14
3	51 ± 7	68 ± 8	170 ± 12
5	56 ± 8	63 ± 9	169 ± 11

α^0^_C_ and α^T^_C_ denote the degree of crystallinity of PHB for the initial (non-annealed) sample and after annealing at 140 °C for 2 h, respectively; T_MP_ is the melting point temperature.

**Table 2:** Kinetic characteristics of diffusion-controlled DPD release from ultrathin PHB fibers.

C_DPD_, wt %	ΔMt/Δt (×10^3^), S^0.5^	R (×10^4^), cm	D_i_ (×10^10^), cm^2^/s	D_m_ (×10^10^), cm^2^/s	F_D_, %	r, Pierson’s Statistics	R^2^ Statistics
1	7.66 ± 0.32	4.2	1.14 ± 0.17	1.05	11.5	0.99155	0.98318
3	7.34 ± 0.26	3.9	0.98 ± 0.14	1.05	28.5	0.99377	0.98758
5	8.43 ± 0.38	4.0	1.19 ± 0.21	1.05	17.0	0.98993	0.97996

C_DPD_, drug loading concentration; R, mean radius of the monofilament; D_i_ and D_m_ are experimental and modeling diffusion coefficients, respectively; F_D_, objective function describing the difference between the modeled and experimental diffusivities; r and R^2^, Pearson correlation coefficient and the coefficient of determination (linear regression), respectively, obtained using OriginLab 2018 software.

## Data Availability

The original contributions presented in this study are included in the article/Supplementary Materials. Further inquiries can be directed to the corresponding authors.

## Supplementary Materials

The following supporting information can be downloaded at: https://www.mdpi.com/article/10.3390/ijms27125189/s1.
