# Supplementary material for: Diffusion-Controlled Drug Release from Electrospun Poly(3-hydroxybutyrate) Fibers with Beaded Architecture: An Experimental and Modeling Study
Source: Int J Mol Sci. 2026 Jun 8;27(12):5189. doi: 10.3390/ijms27125189 (PMC13299927; doi:10.3390/ijms27125189)
Supplement: Supplementary file 1 [file ijms-27-05189-s001.zip › ijms-4321623-supplementary.pdf]

Supplementary

A series of intermediate equations for the solution of diffusional transport in ellipse

Solving equation (2) by separating the variables $\Phi(\xi,\eta) = R(\xi)S(\eta)$ leads to the equations

$$(\xi^2 - 1)\frac{\partial^2 R}{\partial \xi^2} + 2\xi\frac{\partial R}{\partial \xi} - (\lambda\text{-}c^2\xi^2)R = 0 \qquad \text{(S1)}$$

$$(1 - \eta^2)\frac{\partial^2 S}{\partial \eta^2} - 2\eta\frac{\partial S}{\partial \eta} + (\lambda\text{-}c^2\eta^2)S = 0, \qquad \text{(S2)}$$

where λ - is the eigenvalue, emerging due to the separation variables procedure.

Under desorption conditions, for any $t > 0$, the concentration $C(\xi_0, \eta, t)$=0 on the surface of the ellipsoid $\xi_0 = cosh(r_0) = \frac{a}{\mathrm{F}\,0}$. Whence we find for each fixed n the values c_{nk}, as zero values for the function $R_n(c_{nk}, \xi_0) = 0,\ k = 1,2,\dots$.

And for each c_{nk} the eigenvalues $\lambda_n(c_{nk})$ are calculated. The Sturm-Liouville problem has an infinite discrete set of eigen functions $S_n(\ c_{nk}, \eta)$. These functions form a complete orthogonal system $L_2(-1,1)$ - the square-integrable function.

The parity of functions S_n coincides with the parity of the number of zeros by (-1,1), so that for the chosen numbering $S_n(\ c_{nk}, \eta) = (-1)^n\, S_n(c_{nk}, -\eta)$. Under the conditions of our problem, the concentration distribution in the ellipsoid with respect to the z=0 plane must be symmetric.

This condition is satisfied when $S_n(\ c_{nk}, n)$ is an even function, and $n = 0, 2, \dots$.

For different n of equal parity, the functions R_n are orthogonal on [1, +∞).

The coefficients A_{nk} were determined by the evaluation being executed for a system of orthogonal functions. The determinant of the Jacobian $\mathcal{J}$ of the transformation from prolate spheroidal coordinates to Cartesian equals to

$$\det \mathcal{J}\, dr d\theta = {F_0}^3(\xi^2 - \eta^2)\sinh(\mathrm{r})\sin(\theta) dr d\theta = -{F_0}^3(\xi^2 - \eta^2) d\xi d\eta. \quad \text{(S3)}$$

Then,

$$A_{nk} = \frac{1}{B_{nk}}\left(\int_0^1 \int_1^{\frac{a}{\mathrm{F}_0}} C_0(\xi,\eta,0)\,(\xi^2 - \eta^2)\ \ R_n(c_{nk},\xi)\,S_n(c_{nk},\eta) d\xi d\eta\right), \qquad (S4)$$

Where

$$B_{nk} = \int_0^1 \int_1^{\frac{a}{\mathrm{F}_0}} (\xi^2 - \eta^2)\ \ [R_n(c_{nk},\xi) S_n(c_{nk},\eta)]^2 d\xi d\eta. \qquad \text{(S5)}$$
